# Nilotinib impairs skeletal myogenesis by increasing myoblast proliferation

**DOI:** 10.1186/s13395-018-0150-5

**Published:** 2018-02-20

**Authors:** Osvaldo Contreras, Maximiliano Villarreal, Enrique Brandan

**Affiliations:** Departamento de Biología Celular y Molecular and Center for Aging and Regeneration (CARE-ChileUC), Facultad de Ciencias Biológicas, Pontificia Universidad Católica de Chile, Libertador Bernardo O’Higgins 340, 8331150 Santiago, Chile

**Keywords:** Nilotinib, Skeletal muscle, Differentiation, Myoblasts, Proliferation, Tyrosine kinase inhibitors, p38 MAPK, ERK1/2, AKT

## Abstract

**Background:**

Tyrosine kinase inhibitors (TKIs) are effective therapies with demonstrated antineoplastic activity. Nilotinib is a second-generation FDA-approved TKI designed to overcome Imatinib resistance and intolerance in patients with chronic myelogenous leukemia (CML). Interestingly, TKIs have also been shown to be an efficient treatment for several non-malignant disorders such fibrotic diseases, including those affecting skeletal muscles.

**Methods:**

We investigated the role of Nilotinib on skeletal myogenesis using the well-established C2C12 myoblast cell line. We evaluated the impact of Nilotinib during the time course of skeletal myogenesis. We compared the effect of Nilotinib with the well-known p38 MAPK inhibitor SB203580. MEK1/2 UO126 and PI3K/AKT LY294002 inhibitors were used to identify the signaling pathways involved in Nilotinib-related effects on myoblast. Adult primary myoblasts were also used to corroborate the inhibition of myoblasts fusion and myotube-nuclei positioning by Nilotinib.

**Results:**

We found that Nilotinib inhibited myogenic differentiation, reducing the number of myogenin-positive myoblasts and decreasing myogenin and MyoD expression. Furthermore, Nilotinib-mediated anti-myogenic effects impair myotube formation, myosin heavy chain expression, and compromise myotube-nuclei positioning. In addition, we found that p38 MAPK is a new off-target protein of Nilotinib, which causes inhibition of p38 phosphorylation in a similar manner as the well-characterized p38 inhibitor SB203580. Nilotinib induces the activation of ERK1/2 and AKT on myoblasts but not in myotubes. We also found that Nilotinib stimulates myoblast proliferation, a process dependent on ERK1/2 and AKT activation.

**Conclusions:**

Our findings suggest that Nilotinib may have important negative effects on muscle homeostasis, inhibiting myogenic differentiation but stimulating myoblasts proliferation. Additionally, we found that Nilotinib stimulates the activation of ERK1/2 and AKT. On the other hand, we suggest that p38 MAPK is a new off-target of Nilotinib. Thus, there is a necessity for future studies to investigate the long-term effects of TKIs on skeletal muscle homeostasis, along with potential detrimental effects in cell differentiation and proliferation in patients receiving TKI therapies.

**Electronic supplementary material:**

The online version of this article (10.1186/s13395-018-0150-5) contains supplementary material, which is available to authorized users.

## Background

During skeletal muscle development and regeneration, myogenic precursors undergo several cellular and molecular changes to differentiate into multinucleated myofibers [1]. Early events are characterized by the expression of muscle regulatory factors (MRFs) such as MyoD, Myf5, and myogenin. Later, muscle specific genes (i.e., myosin heavy chain) began to be expressed along with the appearance of differentiated myotubes due to myoblasts fusion [2]. In the adult, muscle stem cells (MuSCs) are a reservoir of quiescent myogenic precursors with an essential role in muscle regeneration [3]. Specifically, the ablation of Pax7-satelite cells causes a complete loss of muscle regenerative capacity and fibro-fatty degeneration after acute damage [4, 5]. Fibro-fatty degeneration is also a hallmark of chronic degenerative diseases such as muscular dystrophies (MD), a set of disorders caused by different genetic mutations characterized by progressive muscle weakness and degeneration [6, 7]. The major contributor to ectopic fibro-fatty formation in several muscle disorders is a population of non-myogenic mesenchymal cells, called fibro/adipogenic progenitors (FAPs), that can be identified by the expression of platelet-derived growth factor receptor alpha (PDGFRα) [5, 8–12]. Intriguingly, these cells also have a supportive role during myogenesis and healthy muscle regeneration [6, 10, 13–17].

Numerous experimental approaches have been developed to improve muscle regeneration and reduce fibrosis in MD [18–22]. Nilotinib (Tasigna/AMN107®; Novartis) is a potent and selective tyrosine kinase inhibitor rationally designed as a substitute for Imatinib (Gleevec/STI-571®; Novartis) to overcome resistance and intolerance in patients with chronic myelogenous leukemia (CML) [23]. Nilotinib and Imatinib bind to and stabilizes the inactive conformation of the kinase domains of Abl, c-kit, platelet-derived growth factor receptors (PDGFRα and PDGFRβ), and discoidin domain receptors (DDR-1 and DDR-2) [24]. Both FDA-approved tyrosine kinase inhibitors (TKIs) show potent antifibrotic activity in the liver, skin, and lung from different animal models [25–28]. These studies suggest that TKIs are not only revolutionary anti-proliferative agents but can also target non-malignant disorders and fibrosis [29]. In skeletal muscle, both inhibitors successfully attenuate muscle pathology in several MD mice models [16, 30, 31]. However, the pharmacological blockage of FAP expansion by Nilotinib also reduces myoblast proliferation in a model of acute damage [13]. The authors of this study suggest that there is a non-cell autonomous effect of FAP-restricted expansion on myoblast precursors during normal regeneration. Thus, Nilotinib exhibits detrimental effects on healthy muscle regeneration [13]. However, the potential effects of Nilotinib on skeletal myogenesis have not been directly addressed to date.

The mitogen-activated protein kinase (MAPK) family plays an essential role in transducing the extracellular signals to cellular responses such as proliferation, differentiation, and apoptosis [32]. In addition, ERK1/2 and p38 MAPKs are essential for myoblast proliferation and differentiation [33–35]. The PI3K/AKT signaling pathway is also a major mediator of cell survival through the inhibition of pro-apoptotic proteins and is often stimulated simultaneously with the RAF/MEK/ERK pathway by the same signals [36]. Nilotinib has demonstrated off-target activity against the MEK/ERK pathway in a variety of cancer cell lines carrying RAS mutations, thus driving a paradoxical activation of BRAF and CRAFT [37]. However, to our knowledge, there is no evidence that Nilotinib can interfere directly with p38, ERK1/2, or AKT signaling in non-malignant cells.

Interestingly, a high proportion of CML and gastrointestinal stromal tumor patients have reported some muscle side effects related to TKI treatments. Those negative effects include myalgia, muscle cramps, elevated creatine kinase levels, muscle edema, rhabdomyolysis, and significant restrictions in daily activities. In many cases, these serious side effects can result in the reduction of the indicated dose or in the interruption of treatment [38–40]. Additionally, the lack of muscle histological and in vitro studies during tyrosine kinase inhibitor therapies hinders the evaluation of possible off-target effects in the cells and tissues [38]. These data led to the hypothesis that Nilotinib could potentially have deleterious effects on muscle homeostasis. Here, we investigated the effects of this drug on myogenic differentiation leading to the discovery that Nilotinib is a potent negative modulator of skeletal myogenesis. Nilotinib inhibits myoblast-to-myotube transition, by decreasing the expression of *myogenin* and *MyoD* and reducing myotube formation. This compound also modified myotube-nuclei positioning. In addition, by combining 3D protein structural analysis, protein alignment, and cell-based experiments, we determined that p38 MAPK protein is a novel off-target of Nilotinib. Nilotinib inhibits p38 phosphorylation, while it activates ERK1/2 and AKT signaling pathways in myoblasts. Moreover, we found that Nilotinib induces myoblast proliferation, causing impairments in myoblast cell-cycle withdrawal through both ERK1/2 and AKT pathways.

## Methods

### Reagents

Nilotinib (AMN-107) (CDS023093, Sigma-Aldrich, St. Louis, MO, USA) was reconstituted in DMSO (D2650, Sigma-Aldrich), and cells were treated at final concentrations indicated in the corresponding figures. DMSO was used as a control. 5-Bromo-2′-deoxyuridine (BrdU) (B5002, Sigma-Aldrich) was used in C2C12 myoblasts for 24 h at a final concentration of 10 μM in differentiation medium. 7-Aminoactinomicyn D (7-AAD) was obtained from BioLegend (420403, San Diego, CA, USA) and reconstituted according to the manufacturer’s instructions. The following inhibitors were added to the cell medium 30 min prior Nilotinib treatment: PI3K/AKT inhibitor LY294002 (10 μM) (440202, Merck-Calbiochem, Darmstadt, Germany), the inhibitor of MEK1/2/ERK1/2 kinases UO126 (10 μM) (#9903, Cell Signaling, MA, USA). Cytosine β-D-arabinofuranoside (Ara-C) (100 μM) (C1768, Sigma-Aldrich) was added at days 3 and 4 of C2C12 skeletal muscle differentiation when indicated in the corresponding figures.

### C2C12 myoblast cell line culture

C2C12 myoblasts (American Type Culture Collection, VA, USA) were cultured at 37 °C in 5% CO_2_ in GM; DMEM high glucose (Invitrogen, CA, USA) with 10% fetal bovine serum (FBS) (Hyclone, UT, USA) and supplemented with antibiotics. We induced skeletal muscle differentiation at 80–90% of myoblasts confluence by changing the growth medium to differentiation medium (DMEM high glucose + 2.5% horse serum) [41]. When Nilotinib, UO126, and LY294002 inhibitors were used, the differentiation medium was changed every day along with the compounds. For experiments related to FAK, p38, SAPK/JNK, ERK1/2, and AKT phosphorylation, C2C12 cells were serum-starved for 1 h prior to treatment with Nilotinib.

### Primary muscle cell culture and myotube formation

Primary myoblasts were derived from limb muscles from 2-month-old female WT C57BL/6 (*n* = 3) mice; the protocol was adapted from previous study (Additional file 1: Figure S6) [42]. Briefly, limb muscles were dissected, minced, and subjected to collagenase/dispase (2.5 mg/ml) (C3180, Sigma-Aldrich, USA) digestion for 45 min at 37 °C. The digested tissue was then vortexed followed by filtration through 70-μm-nylon mesh filters (352340, Falcon, Durham, USA). After centrifugation, mono-nucleated cells were suspended in myoblast growth medium, DMEM high glucose supplemented with 10% FBS plus 10% HS, and pre-incubated on 10-cm cell culture dishes for 1.5 h. Non-adherent cells were collected and seeded onto six-well plastic plates coated with 1% gelatin, and another 3 h pre-incubation was performed. The supernatant was discarded, and adherent cells incubated until they reach 50–60% of confluence. Myotube formation was induced by changing growth medium to differentiation medium (DMEM high glucose supplemented with 5% horse serum). Adult primary myoblasts were kept at 37 °C, 8% CO_2_, and 95% humidity. Nilotinib (5 μM) was added at day 0 since differentiation induction and changed every 24 h.

### Protein extraction and western blot analysis

Protein extracts from cells were obtained using RIPA 1× lysis buffer (Cell signaling #9806, MA, USA) plus 1 mM phenylmethylsulfonyl fluoride (Sigma-Aldrich, USA). Then, the cells were sonicated for 10 s and centrifuged at 9000 g. Proteins were quantified with the Micro BCA assay kit, following the manufacturer’s instructions (Pierce, IL, USA). Extracts were subjected to SDS-PAGE electrophoresis in 9% polyacrylamide gels (10% for phospho-histone 3 experiments), transferred to PDVF membranes (Millipore, CA, USA), and probed with primary antibodies: mouse anti-α-tubulin (1:5000) (T5168, Sigma-Aldrich), rabbit anti-phospho-FAK Y397 (1:1000) (#3283, Cell Signaling, MA, USA), rabbit anti-FAK (1:1000) (#sc-558, Santa Cruz, CA, USA), rabbit anti-phospho-p44/42 MAPK (ERK1/2) (1:1000) (#9101S, Cell Signaling), rabbit anti-p44/42 MAPK (ERK1/2) (1:1000) (#9102, Cell Signaling), rabbit anti-phospho-AKT (ser473) (1:1000) (#9271S, Cell Signaling), rabbit anti-Akt (1:1000) (#9272, Cell Signaling), rabbit anti-phospho p38 (Thr180/Tyr182) (1:500) (#9211S, Cell Signaling), rabbit anti-p38 (1:1000) (#9212, Cell Signaling), rabbit anti-phospho-SAPK/JNK (1:1000) (Thr183/Tyr185) (#9251, Cell Signaling), mouse anti-Myosin Skeletal Fast (1:1000) (#M4276, Sigma-Aldrich), mouse anti-GAPDH (1:5000) (#MAB374, Millipore, CA, USA), mouse anti-Pax7-c (1:1000 from concentrate) (Developmental Studies Hybridoma Bank), rabbit anti-myogenin (1:500) (#sc-576, Santa Cruz), rabbit anti-phospho histone 3 (ser28) (1:500) (#07-145, Millipore), Murf1 (c-11) (1:500) (#sc-398608, Santa Cruz), and MAFbx (Atrogin-1) (F-9) (1:500) (#sc-166806, Santa Cruz). Primary antibodies were detected with a secondary antibody conjugated to horseradish peroxidase: mouse anti-goat IgG, #31400; goat anti-rabbit IgG, #31460; and goat anti-mouse IgG, #31430 (1:5000) (Pierce, IL, USA). All immunoreactions were visualized by enhanced chemiluminescence SuperSignal West Dura (34075, Pierce, IL, USA) or SuperSignal West Femto (34096, Pierce, IL, USA) by a ChemiDoc-It HR 410 imaging system (UVP, CA, USA). Western blot densitometry quantification was done using ImageJ software (version 1.46r, NIH, USA). Protein levels were normalized with the levels of the loading control. For phosphorylation studies, myoblasts were serum-starved for 1 h before treatments.

### Crystal violet stain

**Fig. 1:**
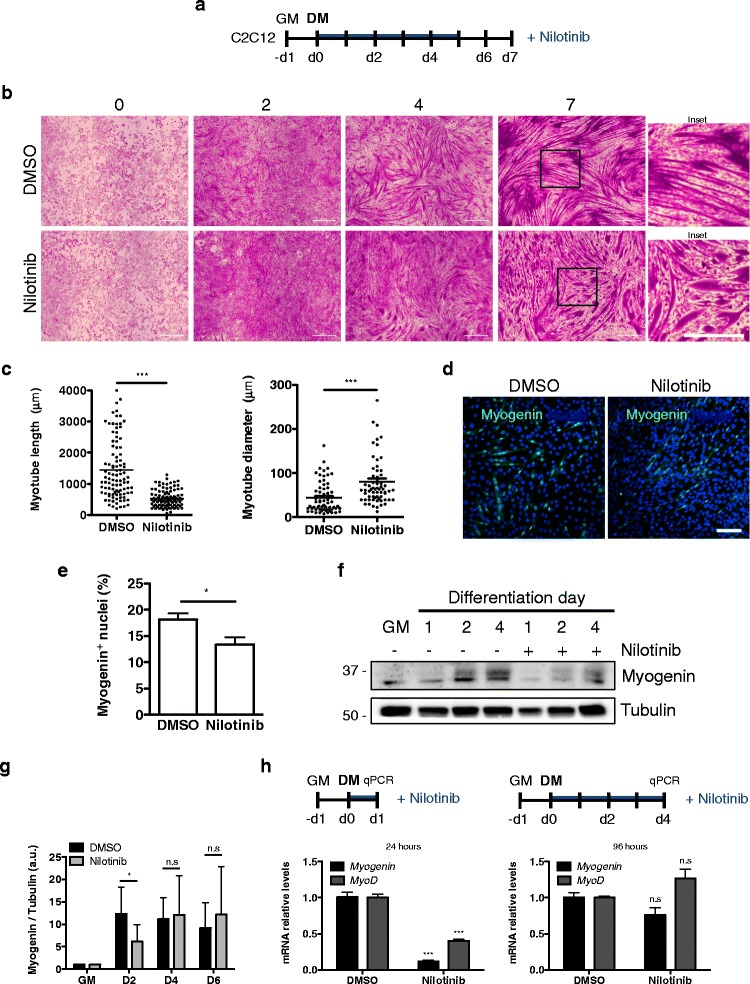
Nilotinib inhibits myogenic differentiation. **a** Outline of C2C12 skeletal muscle differentiation and Nilotinib (5 μM) treatment protocol. DMSO was used as control. **b** Representative crystal violet staining images of a differentiation time-course assay of C2C12 cells. Insets show a higher magnification of D7 myotubes. Scale bars: 500 μm. **c** Quantification (mean ± SEM) of myotube length and diameter at day 7 of differentiation. *n* = 3; ***P < 0.001; DMSO vs Nilotinib; with two-tailed Student’s *t* test. **d** Representative immunofluorescence analysis of C2C12 myoblasts after 48 h of DMSO or Nilotinib treatment in differentiation medium shows nuclear (Hoechst in *blue*) localization of myogenin. Scale bar: 50 μm. **e** Quantification of the percentage (%) of myogenin-positive cells per field after 48 h of DMSO or Nilotinib treatment in differentiation medium. Values correspond to the mean ± SEM (*n* = 3). *P < 0.05; DMSO vs Nilotinib; with two-tailed Student’s *t* test. **f** Representative Western blot analysis that evaluates myogenin expression levels in DMSO or Nilotinib-treated myoblasts during a 4-day skeletal muscle differentiation time course. Tubulin was used as the loading control. *GM* growth medium. **g** Quantification of myogenin expression during a 6-day skeletal muscle differentiation time course. Values correspond to the mean ± SEM. *n* = 6; *P < 0.05, *n.s* non-significant; one-way ANOVA with Bonferroni post-test. **h** *MyoD* and *Myogenin* expression levels were analyzed by quantitative PCR in C2C12 myoblasts after 24 h (left graph) and 96 h (right graph) of treatment in differentiation medium. The values correspond to the mean ± SEM. *n* = 4; ***P < 0.001, *n.s* not significant

The cells were washed twice in cold PBS 1× and then fixed in 100% cold methanol (− 20 °C) for 2 min. After methanol removal, Crystal Violet Staining Solution (0.5% *w*/*v*) (C3886, Sigma-Aldrich, USA) was added and immediately washed with abundant distilled water. Stained cells were imaged in a Nikon Eclipse N600 microscope. We measured myotube length and diameter size in 5 randomly chosen fields. Quantification of Fig. 1c (100 myotubes for length and 65 myotubes for diameter) was done using ImageJ software (version 1.46r, NIH, USA).

### Indirect immunofluorescence

For immunofluorescence analyses, the cells were seeded on 9.2 cm^2^ tissue culture dishes (TPP #93040). At the end of experiments, cells were washed three times with PBS 1×, fixed for 10 min in cold 4% paraformaldehyde, and washed with PBS again. Then, the cells were permeabilized with PBS 1×, 0.1% Triton X-100 for 2 min, blocked for 30 min in blocking buffer (PBS 1× 0.1% Triton X-100 + 1% BSA + 1% fish gelatin) and incubated with the primary antibody overnight: mouse anti-Myosin Skeletal Fast (1:250) (#M4276, Sigma-Aldrich), rabbit anti-Ki67 antibody (1:50) (#15580, Abcam), rabbit anti-myogenin (1:50) (#sc-576, Santa Cruz), supernatant mouse G3G4 anti-BrdU (DSHB Hybridoma Product G3G4). Next, the samples were washed with PBS 1× and incubated for 1 h at room temperature with Alexa Fluor secondary antibodies (1:500 dilution) (Invitrogen, CA, USA). Next, Hoechst 33258 was added for 10 min for staining of nuclei. Cells were washed with PBS 1×, and DAKO fluorescent mounting medium (Dako North America Inc., CA, USA) was added. To stain F-actin Alexa Fluor 568 Phalloidin was added to the cells according to provider’s instructions (#A12380, ThermoFisher, MA, USA). Cells were imaged on a Nikon Eclipse C2 si confocal spectral microscope using NIS-Elements AR software 4.00.00 (build 764) LO, 64 bit. The objectives used were Plan Apo VC 20× DIC N2 NA 0.75, Plan Apo VC 40× OIL DIC N2 NA 1, and Plan Apo VC 60× Oil DIC N2 NA 1.4.

**Fig. 2:**
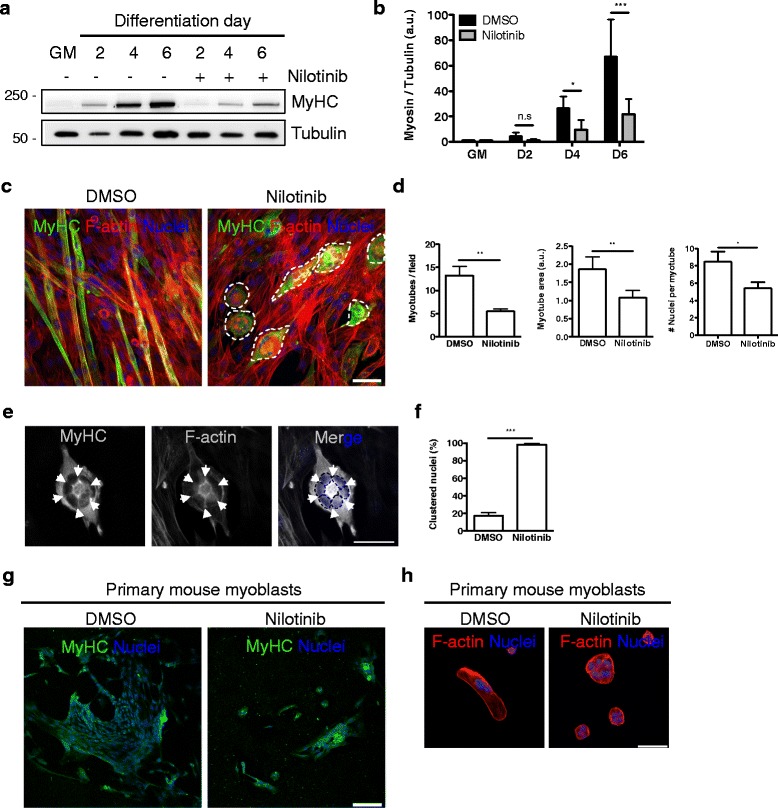
Nilotinib impairs MyHC expression and myotube formation. **a** Representative Western blot analysis, evaluating MyHC expression levels during a 6-day skeletal muscle differentiation time course. Tubulin was used as the loading control. **b** Quantification of six independent experiments, evaluating the expression of MyHC. The values correspond to the mean ± SEM. ***P < 0.001, *P < 0.05, *n.s* non-significant; *n* = 6; one-way ANOVA with Bonferroni post-test. **c** Immunofluorescence analyses showing the expression of MyHC (*green*) and F-actin (*red*) in differentiated myotubes at day 6. Dotted lines mark abnormal clustered myotubes due to Nilotinib treatment. Nuclei were stained with Hoechst (*blue*). Scale bar: 50 μm. **d** Quantification of three representative experiments, evaluating myotube number (left graph), area (center graph), and fusion index (# nuclei per myotube). The values correspond to the mean ± SEM. *n* = 3; **P < 0.005, *P < 0.05 DMSO vs Nilotinib; with two-tailed Student’s *t* test. **e** Representative immunofluorescence images showing ring-shaped clustered nuclei around a single myotube in Nilotinib-treated cells. MyHC (*gray*) and F-actin (*gray*) were stained, and Hoechst (*blue*) was used to identify nuclei. Arrows (*white*) mark intermediate zones in-between individual nuclei. Dotted lines (*black*) mark single nuclei. Scale bar: 50 μm. **f** Quantification of three representative experiments, evaluating the percentage (%) of clustered nuclei (ring-like structure) in DMSO and Nilotinib-treated cells. The values correspond to the mean ± SEM. *n* = 3; ***P < 0.001; DMSO vs Nilotinib; with two-tailed Student’s *t* test. **g** Primary mouse myoblast cells were treated with DMSO or 5 μM Nilotinib. Myogenic differentiation (at day 3 of DM) was assessed by MyHC immunofluorescence. **h** Ring-like structures were always observed when primary mouse myoblasts were differentiated in the presence of Nilotinib. F-actin (*red*) was used to label cell structure. Scale bars: 100 μm (**g**) and 50 μm (**h**)

To quantify myotube number, area, and differentiation index (number of nuclei per myotube), we used MyHC staining of C2C12 cells at day 6 of skeletal muscle differentiation (Fig. 2d). To calculate the myotube area, MyHC stained images were converted to 8-bit, then we applied Huang threshold (B&W) with dark background, and the area was measured using the ImageJ software to analyze particle function (version 1.46r, NIH, USA). To calculate the number of nuclei per myotube, we counted the nuclei on each MyHC^+^ myotube (MyHC^+^-cell with 3 > nuclei was considered a myotube). For myotube number, area and differentiation index counts of 4 to 8 randomly chosen fields were averaged from three independent experiments.

### Immunofluorescence analyses of Ki67, myogenin, and BrdU labeling

The percentages of myogenin-, Ki67-, and BrdU-positive cells were determined using ImageJ software (version 1.46r, NIH, USA) analyze particles function or by counting the positive cells individually per field using the Image J cell counter plugin. Hoechst staining was used to determine the total cell count. For BrdU immunofluorescence, we incubated the PFA-fixed cells with 2N HCl for 30 min at 37 °C. Then, 2N HCl was washed 2 times for 5 min with neutralization 0.1M Borate buffer (pH 8.5). After this procedure, immunofluorescence was performed as described before. For each experimental condition, counts of 6 to 10 randomly chosen fields were averaged for three independent experiments.

### RNA isolation, reverse transcription, and quantitative real-time polymerase chain reaction (RT-qPCR)

Total RNA from cultured cells was isolated using TRIzol (Invitrogen, CA, USA) according to the manufacturer’s instructions. RNA integrity was corroborated as described before [43]. Two microgram RNA was reverse transcribed into cDNA using random primers and M-MLV reverse transcriptase (Invitrogen, CA, USA). RT-qPCR was performed in duplicate with the Eco Real-Time PCR System (Illumina, CA, USA), using primer sets for *myogenin*, *MyoD*, *cyclinD1*, and the housekeeping gene *18s* (used as a reference gene). The ΔΔCt method was used for quantification, and mRNA levels were expressed relative to the mean level of the control condition in each case (DMSO treated cells). We analyzed *myogenin*, *MyoD*, and *cyclinD1* RT-PCR expected product using a 2% agarose gel.

**Table:** 

Gene	Forward primer	Reverse primer
*MyoD*	5’–GCCGCCTGAGCAAAG	5’–CAGCGGTCCAGTGCG
	TGAATG–3’	TAGAAG–3’
*Myogenin*	5’–GTCCCAACCCAGGAG	5’–CCACGATGGACGTAA
	ATCAT–3’	GGGAG–3’
*CyclinD1 (Ccnd1)*	5’-CCCAACAACTTCCTC	5’-TCCAGAAGGGCTTCA
	TCCTG−3’	ATCTG−3’
*18S*	5’–TGACGGAAGGGCA	5’–CACCACCACCCA
	CCACCAG–3’	CGGAATCG–3’

### Computational database, web interface, and protein alignment

Figure 4a image was generated with the DiscoveRx TREEspot™ Compound Profile Visualization Software v4.0 using quantitative affinity data derived from the DiscoveRx KINOMEscan® platform (https://www.discoverx.com/technologies-platforms/competitive-binding-technology/kinomescan-technology-platform). Structural comparisons were performed based on the conformations found in the corresponding ligand–protein crystal structures (PDB entry 3GP0). MAPK11 (p38β) protein structure in a complex with Nilotinib is represented in 3D using JSmol, an open-source Java viewer for chemical structures in 3D (http://www.jmol.org/). The modeled structure and sequence can be downloaded in PDB (PDB ID: 3GP0) and FASTA formats, respectively. The data from figures and tables in the PDB webpage (http://www.rcsb.org/pdb) can be searched and sorted. For protein alignment between human Abl1 (UniProtKB-P00519-1) and human MAPK 11 (p38β) (UniProtKB-Q15759–1), we used the Pairwise Sequence Alignment tool (http://www.ebi.ac.uk/Tools/psa/emboss_needle/). We corroborated the alignment using the RCSB PDB Protein Comparison Tool (http://www.rcsb.org/pdb/home/home.do#Subcategory-analyze_sequences).

### Statistical analysis

Mean and SEM values as well as the number of experiments performed are indicated in each figure. Statistical significance of the differences between the means was evaluated using the one- or two-way analysis of variance test (ANOVA), with Bonferroni post-test, respectively. Two-tailed Student *t* test was performed when two conditions were compared. Differences were considered significant with a *P* value < 0.05. Data was collected in Microsoft Excel (Redmond, WA, USA), and statistical analysis was performed using Prism 5 software (Graphpad, CA, USA).

## Results

### Nilotinib inhibits skeletal muscle differentiation and MRF expression

**Fig. 3:**
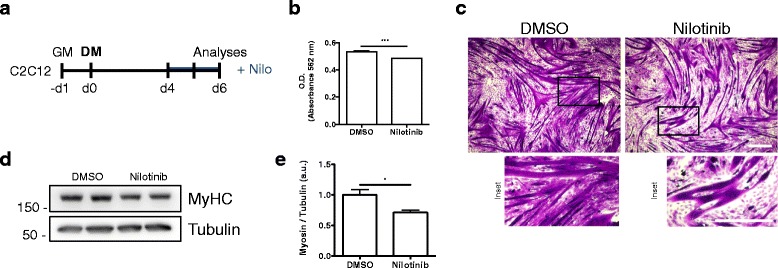
Nilotinib late treatment impairs terminal differentiation. **a** Outline of C2C12 skeletal muscle differentiation and late Nilotinib treatment protocol. *Nilo*, Nilotinib. **b** Total protein content was determined using the BCA assay and O.D. obtained in control of Nilotinib-treated cells. *n* = 3; ***P < 0.001; DMSO vs Nilotinib; with two-tailed Student’s *t* test. **c** Representative crystal violet staining images. Insets show a higher magnification of D6 myotubes. Scale bars: 500 μm. **d** Western blot analysis of three representative experiments to evaluate the total amount of MyHC. Tubulin was used as the loading control. **e** Quantification of three independent experiments to evaluate MyHC expression. Values correspond to the mean ± SEM. *P < 0.05; *n* = 3; with two-tailed Student’s *t* test

It has been suggested that Nilotinib does not affect in vitro proliferation of satellite cells [13], but the function of this tyrosine kinase inhibitor has not been tested during skeletal muscle differentiation yet. To carry out this study, we used the well-established murine C2C12 myoblast cell line and designed a protocol for Nilotinib treatment (Fig. 1a). Next, we evaluated the differentiation of myoblasts into myotubes using crystal violet staining (Fig. 1b). In our hands, myotube formation began at days 2 to 4 upon the addition of differentiation medium, and differentiated myotubes are observed from days 4 to 7 (Fig. 1b, upper panels). When Nilotinib was added myotube differentiation was inhibited. This effect was evident from day 4 after the induction of differentiation (Fig. 1b). Nilotinib treatment alters myotubes elongation, resulting in shortened and thickened myotubes (Fig. 1b, insets; Fig. 1c). Then, we evaluated the effect of Nilotinib on the expression of myogenin. We found that treatment with Nilotinib reduces the percentage of myogenin-positive cells 48 h after C2C12 differentiation (Fig. 1d, e). In addition, Nilotinib treatment significantly delayed the expression of this myogenic regulatory factor (Fig. 1f, g). The results presented above suggest that Nilotinib has a transient impact reducing myogenin expression levels. To confirm this observation, we evaluated myogenin mRNA expression at two different time points (Fig. 1h). Consistent with our results, Nilotinib reduces myogenin mRNA levels at 24 h but not at 96 h of treatment (Fig. 1h). Contrary to what we found with myogenin expression, Nilotinib does not affect Pax7 protein levels during myoblast differentiation (Additional file 2: Figure S1). Overall, this data indicates that Nilotinib inhibits the expression of myogenin and myoblasts-to-myotube differentiation, while Pax7 levels are unaffected during skeletal myogenesis. Figure 2a shows that Nilotinib also inhibits the expression of MyHC during differentiation. Remarkably, the expression of MyHC in Nilotinib-treated cells at differentiation day 6 resembles the expression between differentiation days 2 and 4 in DMSO-treated cells (Fig. 2a, b). Hence, Nilotinib significantly inhibits skeletal myogenesis and therefore delays MyHC expression. Microscopy analyses reveal that Nilotinib impairs the formation of MyHC^+^-myotubes evaluated at day 6 after the induction of differentiation, reducing myotube number, area, and fusion (Fig. 2c, d). In addition, Nilotinib alters nuclei positioning during skeletal muscle differentiation (Fig. 2e). In control myotubes, the nuclei show the characteristic distribution along the longitudinal axis, being the diameter shaft constant throughout the cell (Fig. 2c). In Nilotinib-treated cells, the majority of myotubes maintains a bipolar shape but appears shorter than controls, with multiple nuclei aggregating in the center of the enlarged cell body (Fig. 2c). The shaft shrinks rapidly from the center, leaving a very thin cell shaft with tips at both ends, as shown in Fig. 2e. As a result, the nuclei from the resulting myotubes form a clustered ring-like structure that cannot be resolved. No fully elongated myotubes are observed in the Nilotinib-treated cultures (Fig. 2e, f). To further confirm our results, we isolated primary adult mouse myoblasts from hindlimb muscles of C57BL/6 (wild-type) mice. Myogenic differentiation was also inhibited by Nilotinib treatment in primary myoblasts (Fig. 2g), and ring-like structures were similar to those found in Nilotinib-treated C2C12 myoblasts (Fig. 2h). Hence, treatment of myoblasts induced to differentiate with Nilotinib inhibits the formation and maturation of myotubes, impairing nuclei positioning and reducing MyHC expression. It is known that myoblasts and myotubes respond differently to various stimuli [44], demonstrating the need to distinguish between pre-differentiation and post-differentiation effects of Nilotinib. Thus, in order to determine whether Nilotinib could be causing late negative effects on myoblast differentiation, we established a protocol where cells were treated late during their differentiation (Fig. 3a). Nilotinib treatment during days 4 to 6 after differentiation also causes negative but less pronounced effects during myogenesis, reducing total protein content (Fig. 3b), myotube maturation (Fig. 3c), and MyHC expression (Fig. 3d, e). These results demonstrate that Nilotinib impairs the in vitro myogenic process even when added at later stages.

### Nilotinib treatment does not induce myoblast apoptosis

Nilotinib induces apoptosis of fibro/adipogenic progenitors in skeletal muscle and exerts anti-neoplastic activity in several types of cancer. Therefore, we evaluated whether Nilotinib affects myoblast survival, hence disturbing skeletal muscle differentiation. We used 7-amino-actinomycin D (7-AAD) fluorescence staining and flow cytometric analysis to assess viability. 7-AAD binds to the DNA of death cells, but intact cells exclude it. Our analyses show that Nilotinib treatment for 24 h does not increase 7-AAD incorporation and fluorescence, compared to control cells (Additional file 3: Figure S2A, B), suggesting that Nilotinib does not impair myoblasts survival.

### p38 MAPK is a novel off-target of the tyrosine kinase inhibitor Nilotinib

**Fig. 4:**
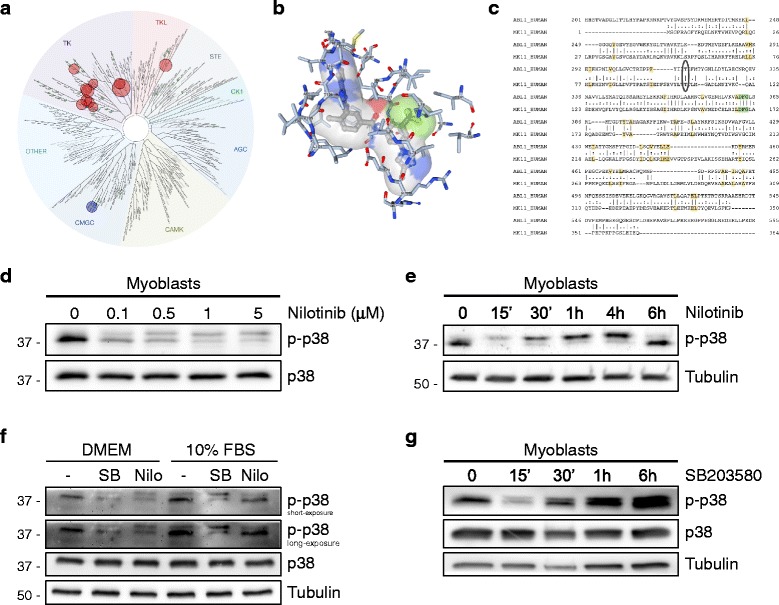
Nilotinib inhibits p38 MAPK in myoblasts. **a** Analysis of Nilotinib binding across the kinome. Interaction map showing a circular representation of the kinase family tree (*KINOMESCAN*, https://www.discoverx.com/technologies-platforms/competitive-binding-technology/kinomescan-technology-plataforma). This map describes quantitatively the interaction patterns of Nilotinib using a 100-nM affinity cutoff (Kd). The blue circle labels the interaction of Nilotinib with the p38 MAPK family. **b** Crystal structure of human MAPK11 (p38β) in complex with Nilotinib. **c** Alignment of human Abl (upper) and MAPK11 (p38 beta) (lower) aminoacid sequences that are part of the SH-1 kinase domains. Amino acids with “|” are structurally equivalent and identical residues, “:” are structurally equivalent and similar residues, “.” are structurally equivalent, but not similar residues. The threonine gatekeeper residues (*boxed*), DFG motif (*green*), and conserved hydrophobic interactions (*yellow*) are shown. **d** The myoblasts were treated with increasing concentrations of Nilotinib for 30 min. Cell lysates were collected for Western blotting of phospho-p38 and total p38. **e** Western blots showing phospho-p38 levels in myoblasts after Nilotinib (5 μM) treatment at different time points. Tubulin was used as loading control. **f** Representative Western blot of myoblasts that evaluates p38 phosphorylation using SB203580 (SB) (5 μM) p38 inhibitor and Nilotinib. Both treatments were performed for 30 min. Total p38 and tubulin were used as loading controls. **g** Western blots showing phospho-p38 levels in myoblasts after SB203580 treatment at different time points. Total p38 and tubulin were used as loading controls

Next, we hypothesized that Nilotinib could be disrupting signaling pathways involved in myoblast proliferation and survival. To define the interaction patterns of Nilotinib quantitatively across the kinome, we calculated individual selectivity scores, using a 100-nM affinity cutoff (Kd), for each of the major kinase groups [45]. As shown in Fig. 4a, Nilotinib has major selectivity for tyrosine kinases (TK), but it can also interact with the CMGC kinase family. Nilotinib exhibits binding with p38α and p38β, both members of the mitogen-activated protein kinase (MAPK) family but not with p38δ or p38γ (Fig. 4a) [45]. Accordingly, we used a homology model for Nilotinib bound to p38β MAPK generated based on the X-ray structure of a complex between Nilotinib and human MAPK11 (PDBID: 3GP0). This model proposes a strong binding between p38 and Nilotinib (Fig. 4b) because it suggests that p38 can accommodate Nilotinib in a similar way to that seen in the complexes it forms with Abl [46]. This observation, although not conclusive, supports the notion that p38 MAPK possesses high amino-acid sequence homology with Abl, both having a threonine “gatekeeper” residue and a small lipophilic residue (Ala) preceding the “Asp-Phe-Gly (DFG)” motif (Fig. 4c). Consistent with these results, we found that Nilotinib inhibits basal p38 phosphorylation at Thr180/Tyr182 in myoblast in the nanomolar range (Fig. 4d). Moreover, Nilotinib treatment causes early inhibition of p38 MAPK phosphorylation, observed as early as 15–30 min and up to 1 h after Nilotinib treatment (Fig. 4e). Then, p38 phosphorylation returns to basal levels. Furthermore, Nilotinib effectively blocked serum-induced p38 phosphorylation in myoblasts, while SB203580 (the canonical p38 inhibitor) also inhibited p38 phosphorylation as previously reported (Fig. 4f) [47]. However, due to antibody specificity, we were unable to distinguish between the p38α, p38β, or p38γ. In order to compare the transient reduction of p38 phosphorylation by Nilotinib, we treated myoblasts with SB203580 during a 6-h time course. The SB203580 p38 inhibitor showed an identical effect as Nilotinib, reducing p38 phosphorylation levels at early times (Fig. 4g). Thus, it is likely that p38 MAPK represents a new physiologically relevant target of Nilotinib.

### Nilotinib induces ERK1/2 and AKT phosphorylation in myoblasts

**Fig. 5:**
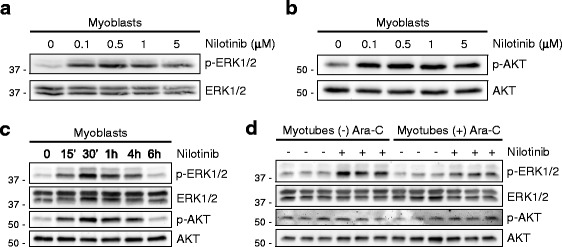
Nilotinib activates ERK1/2 and AKT pathways in myoblasts. Representative Western blot analyses from three independent experiments to evaluate the phosphorylation of (**a**) p44/42 MAPK (ERK1/2) (Thr202/Tyr204) and (**b**) AKT (S473) in myoblasts after Nilotinib treatment at different concentrations. Total ERK1/2 and AKT were used as loading controls. **c** Representative Western blots analysis from three independent experiments that evaluate the phosphorylation of ERK1/2 and AKT in myoblasts after Nilotinib treatment at different time points. Total ERK1/2 and AKT were used as loading controls. **d** Representative Western blots analysis from three independent experiments that evaluate the phosphorylation of ERK1/2 and AKT in 7 days myotubes. Ara-C non-treated and Ara-C-treated myotubes are shown. Nilotinib treatment was performed for 1 h. Total ERK1/2 and AKT were used as loading controls

Next, we evaluated the phosphorylation of ERK1/2 (Thr202/Tyr204) and AKT (Ser473) after Nilotinib treatment. Contrary to the effects on p38, we found that Nilotinib stimulates basal ERK1/2 and AKT phosphorylation in myoblasts in the nanomolar range (Fig. 5a, b). Nilotinib gradually induces ERK1/2 and AKT phosphorylation from 15 min to 1 h returning to basal levels 6 h post-treatment (Fig. 5c). Additional file 4: Figure S3 shows the basal phosphorylation of p38, ERK1/2, and AKT pathways during a 6-day time course of C2C12 differentiation. The p38 phosphorylation is reduced while ERK1/2 and AKT are early activated upon differentiation medium addition (Additional file 4: Figure S3). Nilotinib-induced phosphorylation of ERK1/2 and AKT was fully blocked by inhibitors UO126 and LY294002, respectively (Additional file 5: Figure S4A).

In addition, it has been reported that Nilotinib causes glioblastoma cell invasion through the off-target activation and phosphorylation of focal adhesion kinase (FAK) [48]. FAK is the major downstream target of ECM-integrin signaling and essential in regulating myoblast proliferation and differentiation [41, 49]. Hence, we tested whether Nilotinib could affect FAK activation in myoblasts. Additional file 5: Figure S4B shows that 1-h of Nilotinib treatment does not alter FAK phosphorylation on tyrosine 397. We also tested whether Nilotinib could affect other kinases such as SAPK/JNK, but we did not find changes in SAPK/JNK phosphorylation under the same conditions (Additional file 5: Figure S4B).

To determine whether activation of the ERK1/2 and AKT pathways by Nilotinib occurred at different stages of muscle differentiation, we compared the effect of Nilotinib on myoblasts and differentiated myotubes. Whereas Nilotinib induced activation of ERK1/2 and AKT in myoblasts, we found significant lower levels of ERK1/2 phosphorylation in fully differentiated myotubes (Ara-C-treated) (Fig. 5d). Contrary to myoblasts, Nilotinib-triggered AKT activation was not seen in myotubes. Notably, the activation of the ERK1/2 pathway was only noticeable when we induced skeletal muscle differentiation in the absence of Ara-C, suggesting that reserve myoblasts could account for the activation of ERK1/2 (Fig. 5d). On the other hand, we were unable to detect p38 phosphorylation in myotubes (data not shown). Thus, the positive regulatory effect of Nilotinib on the ERK1/2 and AKT pathways seems to be specific to undifferentiated muscle cells. Altogether, these data demonstrate that Nilotinib induces ERK1/2 and AKT activation in myoblasts, both key downstream regulators of myoblast proliferation and differentiation.

### Nilotinib-induced myoblast proliferation is dependent on the activation of the ERK1/2 and AKT pathways

**Fig. 6:**
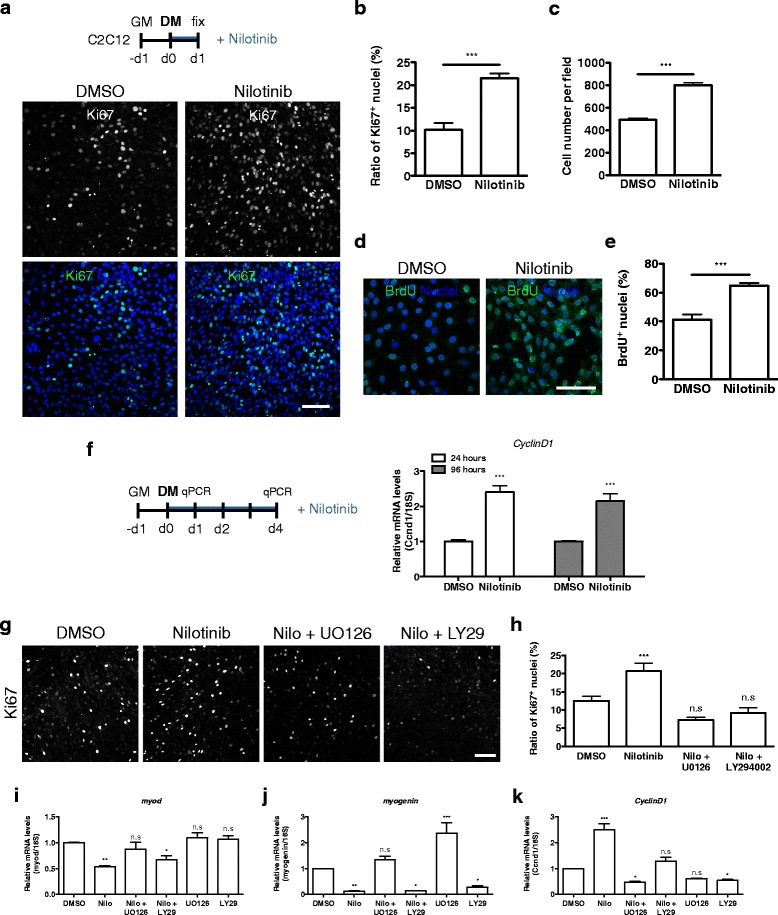
Nilotinib induces myoblast proliferation through ERK1/2 and AKT pathways. **a** Outline of C2C12 skeletal muscle differentiation and 24 h Nilotinib treatment protocol (upper). Immunofluorescence analyses of the proliferation marker Ki67 (*gray* upper panels; *green* lower panels) in C2C12 myoblasts after 24 h of DMSO or Nilotinib (5 μM) in differentiation medium. Hoechst (*blue*) was used to stain nuclei (lower). Scale bar: 50 μm. **b** Evaluation of proliferation as the percentage (%) of Ki67-positive cells per field is shown. The values correspond to the mean ± SEM (*n* = 4). ***P < 0.001; DMSO vs Nilotinib; with two-tailed Student’s *t* test. **c** Quantification of total cell number per field is shown. The values correspond to the mean ± SEM. ***P < 0.001; DMSO vs Nilotinib; *n* = 4; with two-tailed Student’s *t* test. **d** Immunofluorescence analysis of C2C12 myoblasts after 24 h in differentiation medium shows nuclear (Hoechst in *blue*) localization of BrdU proliferation marker. Scale bar: 50 μm. **e** Quantification of proliferation as the % of BrdU^+^-cells. The values correspond to the mean ± SEM (*n* = 3). ***P < 0.001; DMSO vs Nilotinib; with two-tailed Student’s *t* test. **f** Outline of C2C12 skeletal muscle differentiation and 24 or 96 h Nilotinib treatment protocol. *CyclinD1* expression levels were analyzed by quantitative PCR in C2C12 myoblasts after 24 and 96 h treatments in differentiation medium. Values correspond to the mean ± SEM. *n* = 4; ***P < 0.001, **P < 0.005, *P < 0.05, *n.s* not significant. **g** Representative images of Ki67 immunofluorescence in myoblasts treated for 24 h with DMSO, Nilotinib, Nilo (Nilotinib)+UO126, and Nilo + LY29 (LY294002) in differentiation medium. **h** Quantification of three independent experiments, evaluating proliferation as the % of Ki67^+^-cells per field. The values correspond to the mean ± SEM. ***P < 0.001, **P < 0.005, *n.s* non-significant; *n* = 3. One-way ANOVA with Bonferroni post-test. **i** *MyoD*, **j** *myogenin*, and **k** *cyclinD1* expression levels were analyzed by quantitative PCR in C2C12 myoblasts after 24 h of treatments in differentiation medium. Values correspond to the mean ± SEM. ***P < 0.001, **P < 0.005, *P < 0.05, *n.s* not significant; *n* = 4. One-way ANOVA with Bonferroni post-test

To determine whether Nilotinib has an effect on myoblast cell cycle withdrawal, we used immunofluorescence analyses to evaluate proliferation of Nilotinib-treated cells 24 h post-differentiation induction. Our data show that Nilotinib increases myoblasts proliferation twofold, as determined by the percentage of Ki67-positive myoblasts (Fig. 6a, b). Next, we found that Nilotinib increases the total number of myoblasts by 60% after 24 h of treatment (Fig. 6c). To confirm our results, we evaluated BrdU incorporation after a 24-h treatment with Nilotinib (Fig. 6d). Nilotinib significantly increases the percentage of BrdU^+^-cells in differentiation medium by 50%, compared to control cells (Fig. 6e). MTT assays further confirmed our results (Additional file 6). Nilotinib significantly increases myoblasts proliferation rate at the different concentrations used (Additional file 7: Figure S5A). Then, we evaluated mRNA expression of *CyclinD1* at two different time points (Fig. 6f). Consistent with our previous results, Nilotinib treatment induces *CyclinD1* mRNA levels at both time points (Fig. 6f). Altogether, these data demonstrated that Nilotinib promotes myoblasts proliferation during muscle differentiation. The results presented above suggest that ERK1/2 and AKT activation gated by Nilotinib could be impairing cell cycle withdrawal in myoblasts. To test this hypothesis, we inhibited ERK1/2 and PI3K/AKT pharmacologically along with Nilotinib treatment and analyzed myoblast proliferation using Ki67 immunofluorescence. The co-treatment of Nilotinib with the MEK1/2 inhibitor UO126 or the PI3K/AKT inhibitor LY294002 completely abrogated the increase of proliferation induced by Nilotinib (Fig. 6g, h). Additionally, western blot analyses show that Nilotinib increases histone 3 (Ser28)-phosphorylation, a classical marker of mitosis. Thus, our results strongly suggest that Nilotinib induces myoblast proliferation via the activation of the ERK1/2 and PI3K/AKT signaling pathways, thus causing early defects on cell cycle withdrawal and skeletal myogenesis.

### Participation of ERK1/2 and AKT in Nilotinib-inhibited muscle differentiation

Our previous results indicate that Nilotinib was responsible for the induction of ERK1/2 and AKT phosphorylation in myoblasts. Therefore, it should be possible to prevent this activation and to reverse skeletal muscle differentiation and proliferation defects using ERK1/2 and AKT inhibitors along with Nilotinib in myoblasts. Thus, we evaluated the expression of two specific muscle genes *myogenin*, *MyoD*, and the proliferation gene *cyclind1*. In accordance with our previous observations, we found that Nilotinib reduces mRNA levels of both MRFs *MyoD* and *myogenin* (Fig. 6i, j). These effects were dependent on ERK1/2 but not on AKT activation since only co-treatment with UO126 fully blocked the reduction of MRF expression (Fig. 6i, j). In addition, Nilotinib-induced *cyclind1* mRNA levels were completely inhibited by UO126 and LY294002 (Fig. 6k), supporting the role of both ERK1/2 and AKT pathways on Nilotinib-gated proliferation, as shown by previous results. Altogether, our data suggest that the anti-myogenic effects of Nilotinib are ERK1/2 dependent. On the other hand, the proliferation triggered by Nilotinib is both ERK1/2 and AKT dependent.

### Nilotinib affects differentiated myotube proteostasis and MyHC expression

**Fig. 7:**
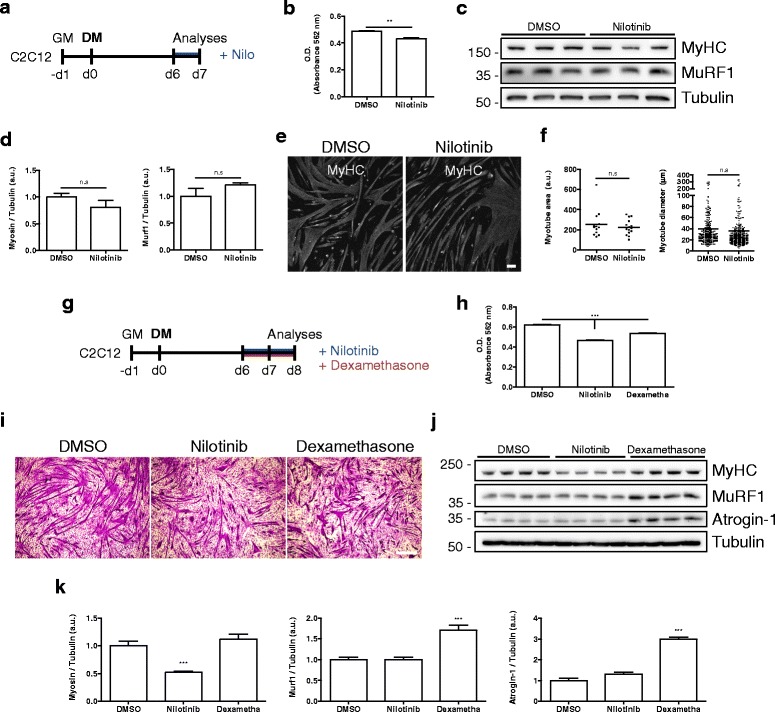
Nilotinib effects on differentiated myotubes. **a** Outline of the 24 h Nilotinib (Nilo) treatment protocol on day 6 C2C12-differentiated myotubes. **b** Total protein content was determined using BCA assay and O.D. obtained in control of Nilotinib-treated cells. *n* = 3; ***P < 0.001; DMSO vs Nilotinib; with two-tailed Student’s *t* test. **c** Western blot analysis of three representative experiments, evaluating the total amount of MyHC and MuRF1. Tubulin was used as the loading control. **d** Quantification of three independent experiments to evaluate MyHC and Murf1 expression. The values correspond to the mean ± SEM. *n.s* non-significant; *n* = 3; with two-tailed Student’s *t* test. **e** Immunofluorescence analyses showing the expression of MyHC (*gray*) in differentiated myotubes at day 7. Scale bar: 100 μm. **f** Quantification of three representative experiments, evaluating myotube area (left graph) and diameter (right graph). The values correspond to the mean ± SEM. *n.s* non-significant; *n* = 3; DMSO vs Nilotinib; with two-tailed Student’s *t* test. **g** Outline of 48 h Nilotinib and dexamethasone treatment protocol on day 6 C2C12-differentiated myotubes. Both compounds were added at 5-μM final concentration. **h** Total protein content was determined using BCA assay and O.D. obtained in control Nilotinib- or dexamethasone-treated cells. *n* = 3; ***P < 0.001; DMSO vs Nilotinib and DMSO vs dexamethasone (Dexametha); One-way ANOVA with Bonferroni post-test. **i** Representative images of crystal violet staining on day 8 myotubes. Scale bar: 500 μm. **j**, **c** Western blot analysis of four representative experiments, evaluating the total amount of MyHC, MuRF1, and Atrogin-1. Tubulin was used as the loading control. **k** Quantification of four independent experiments to evaluate MyHC, Murf1, and Atrogin-1 expression. Values correspond to the mean ± SEM. ***P < 0.001; *n* = 4. One-way ANOVA with Bonferroni post-test

Several chemotherapeutic drugs cause muscle wasting [50, 51]. Muscle RING finger-1 (MuRF1) and muscle atrophy F-box (MAFbx; also known as atrogin-1) are muscle-specific E3 ubiquitin ligases that are increased in skeletal muscle under atrophy-inducing conditions, such as immobilization, denervation, hindlimb unloading, and dexamethasone treatment, among others [52]. Therefore, we evaluated whether Nilotinib induces atrophy on differentiated myotubes. To address this question, we treated differentiated myotubes (day 6) for 24 h (Fig. 7a) or 48 h (Fig. 7g); 24-h Nilotinib-treated myotubes exhibit reduced total protein content (Fig. 7b), but not altered in MyHC expression (Fig. 7c, d). Nilotinib neither affects MuRF1 protein expression after 24 h of treatment (Fig. 7c, d [51]) nor alters myotube area or diameter (Fig. 7e, f). Additionally, when differentiated myotubes were treated twice with Nilotinib for 48 h, total protein content was significantly reduced (Fig. 7h). Similar results were obtained with dexamethasone, a well-known atrophy inducer, showing reduced protein content after 48 h of treatment (Fig. 7h) [53]. Then, we analyzed the morphology of day 8 myotubes using crystal violet staining under the same conditions described before. Importantly, both treatments dramatically affected myotube diameter and elongation after 48 h (Fig. 7i). Moreover, MyHC expression was significantly reduced after Nilotinib but not upon dexamethasone treatment (Fig. 7j, k). Next, we sought to evaluate MuRF1 and Atrogin-1 expression. Dexamethasone successfully induced MuRF1 and Atrogin-1 protein levels (Fig. 7j, k). However, Nilotinib did not induce MuRF1 or Atrogin-1 expression, compared to control myotubes (Fig. 7j, k). Overall, these results although not conclusive suggest that Nilotinib might be causing muscle atrophy through a MuRF1/Atrogin-1-independent mechanism.

## Discussion

Wide ranges of cellular events are modulated by protein tyrosine kinases (PTKs) and are dependent on receptor and non-receptor tyrosine kinase signaling pathways. PTKs participate in proliferation, metabolism, differentiation, and apoptosis under normal and pathological conditions, thus a plethora of studies suggest that TKIs have potential use in many non-malignant diseases [54]. Since several non-malignant diseases have deregulated PTK signaling, and tyrosine kinases are simultaneously activated, TKIs are promising multi-component therapeutic targets not only related to cancer research but also to treat other non-malignant but proliferative disorders including cardiac hypertrophy, pulmonary hypertension, atherosclerosis, rheumatoid disorders, glomerulonephritis, and lung, liver, and muscle fibrosis [54]. In skeletal muscle, Nilotinib strongly blocks PDGFRα and TGF-β pathways, both potent drivers of fibrosis in chronically damaged muscles [16, 55].

Here, we showed that the second-generation TKI Nilotinib inhibits skeletal muscle differentiation. Nilotinib is a negative modulator of skeletal myogenesis, thus alters the expression of both myogenic transcription factors myogenin and MyoD, and reduces myotube number, fusion, and MyHC expression. Also, Nilotinib impairs the normal nuclear positioning in myotubes during myoblast-to-myotube transition. A surprising result of this study is that Nilotinib stimulates myoblast proliferation by simultaneously activating ERK1/2 and AKT. These data provide the first evidence that Nilotinib is a proliferative inductor in non-malignant skeletal muscle cells. In addition, Nilotinib inhibits basal and serum-stimulated phosphorylation of p38 MAPK. Hence, demonstrating that p38 MAPK is a novel and specific off-target of this drug. Notably, the effects described here were induced at clinically relevant drug concentrations [37].

On the other hand, the recent work by Fiore et al. 2016 does not find defects in MyoD-positive myoblast proliferation caused by Nilotinib treatment; thus, our results seem to be in conflict with the results of this group [13]. This disparity could stem from differences in our experimental settings. First, they used isolated myofibers and their associated MuSCs, while we used the C2C12 myoblast cell line. Second, we evaluated myoblast proliferation upon differentiation induction for 24 h, and they did it in MuSCs (in a quiescent state) for 48 h. Overall, future studies are needed to corroborate these results, which underline the need for studying the role of other TKIs on skeletal myogenesis. The results shown here suggest that Nilotinib could also be impairing in vivo myoblast differentiation and therefore healthy muscle regeneration during acute damage. Due to the pleiotropic effects TKIs have on cells, addressing this question on models of acute or chronic muscle damage might present difficulties.

**Fig. 8:**
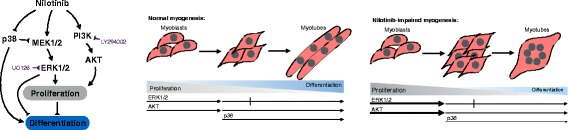
Model of Nilotinib effects on myoblasts and skeletal myogenesis. Nilotinib binds to and inhibits p38 MAPK. Consequently, Nilotinib activates the MEK/ERK proliferation signaling inhibiting myoblast differentiation. Simultaneously, Nilotinib stimulates the AKT survival pathway. In addition, ERK1/2 and AKT stimulation is required for Nilotinib-induced myoblast proliferation. The stimulation of proliferation and the anti-myogenic effects of Nilotinib through the perturbation of p38, ERK, and AKT signaling pathways are illustrated

The Raf/MEK/ERK pathway is known to stimulate cell survival responses by promoting cell cycle progression and proliferation [32]. Importantly, p38 MAPK inhibition during skeletal muscle differentiation induces cell proliferation and inhibits myoblasts differentiation by promoting ERK1/2 activation [47]. Thus, our results showing the simultaneous activation of ERK1/2 and AKT along with the reduction of p38 activity caused by Nilotinib could be part of the same proliferative survival signaling in myoblasts. Moreover, it has been demonstrated that p38 counteracts ERK1/2 and AKT pathways as part of the same pro-survival pathway [32, 47]. Notably, the signaling pathways involving ERK1/2, PI3K/AKT, and p38 control MyoD and myogenin activity [34]. This mechanism might explain the Nilotinib-mediated suppression of the MRFs described in this study. However, further studies are needed to determine the molecular mechanism involved in MyoD and myogenin repression caused by Nilotinib and the molecular consequences of this repression. These data establish that p38/ERK1/2/AKT pathways play a key role in Nilotinib-mediated responses, and therefore, we propose the following model. We postulate that p38 inhibition in parallel with ERK1/2 and AKT over-activation leads to myoblast proliferation due to impaired cell cycle withdrawal and therefore to skeletal myogenesis inhibition (Fig. 8).

One additional mechanism that could explain the Nilotinib-mediated effects described in this work is the fact that Abl, a known non-receptor tyrosine kinase inhibited by Nilotinib, promotes p38 MAPK activation. Abl-silenced C2C12 myoblasts showed lower p38 phosphorylation levels after 2 days in differentiation medium [56]. That study along with our work also opens the possibility that Nilotinib represses p38 activity by two different mechanisms: (1) by directly binding and inhibiting p38 and (2) by binding and inhibiting Abl, thus indirectly inhibiting p38 MAPK. Further studies are needed to distinguish between both possible mechanisms. Another study has shown that p38α is rate limiting for muscle differentiation whereas p38β is not in primary myoblast obtained from knockout mice [57]. Therefore, the inhibition of skeletal myogenesis seen with Nilotinib is likely to occur through the inhibition of p38α instead of by acting on other p38 MAPK subunits. Since the p38 MAPK signaling pathway has fundamental roles on the regulation of muscle stem cell functions is important to elucidate the molecular regulation of p38 by Nilotinib and to evaluate the extent of this regulation on muscle stem cell biology [58].

Additionally, we found that Nilotinib reduces myotube protein content and downregulates MyHC expression. This observation is consistent with the notion that many patients receiving chemotherapeutic drugs develop muscle atrophy. Paradoxically Nilotinib does not induce the two-specific muscle atrophy-related genes MuRF1 or Atrogin-1, when compared to dexamethasone treatment. It has been shown that TKIs impair autophagy [59], which has a crucial role in skeletal muscle tissue homeostasis [60]. Thus, we speculate that one additional mechanism of Nilotinib action in skeletal muscle cells could be impairing autophagy in myoblasts, which could be the cause of the differentiation defects described in our work. Additional experiments are needed to address these questions. Regardless of the mechanistic details, specific inhibition of PTKs by TKIs may be of therapeutic concern in the case of muscle-wasting disorders.

In skeletal tissue, there are two controversial works showing the effects of Nilotinib in bone cells and CML patients. It has been described that Nilotinib can potently inhibit osteoblast proliferation, and in patients, it decreases bone turnover and alters calcium metabolism. While this research group found that Nilotinib has a neutral or inhibitory effect on murine osteoblast differentiation [61], another group showed that it markedly suppresses the osteoblastic differentiation of human mesenchymal stromal cells [55]. Overall, these observations together with our work point out the necessity to further understand the role of Nilotinib off-target activity in non-malignant cells.

It is important to mention that in our study, we have not assessed the classical tyrosine kinases involved in proliferation induction and differentiation arrest described above. Nilotinib potently inhibits ABL kinases, PDGFR, BCR-ABL, KIT, and DDR1/2, all of them associated with cell proliferation and differentiation [25, 29, 62]. Additionally, Nilotinib has been reported to stimulate other kinases, such as p130Cas, FAK, and Paxillin in glioblastoma cells independently of its known targets Abl and PDGFR [48]. Off-target activity of Nilotinib has also been described for the MEK/ERK pathway in several cancer cells [37]. In our work, using different approaches, we described that p38 MAPK is an off-target of Nilotinib in myoblasts. Thus, future studies are needed to find other specific target(s) affected by TKIs during skeletal myogenesis. We propose that Nilotinib could be affecting several kinases at the same time due to its target specificity [46]. Hence, the dissection of the molecular pathways that involve and are affected by TKIs is of fundamental importance for understanding their effects on patients.

Finally, our results show potential unexpected adverse effects of Nilotinib treatment on myoblasts. This drug could be a significant contributor to the muscle homeostasis defects reported by cancer patients receiving TKIs. Therefore, there is a necessity for screening the effects of TKIs on cell homeostasis and to search for disease-specific TKIs, thereby avoiding their undesirable effects in the treatment of cancer and other benign-proliferative disorders.

## Conclusions

Our studies indicate that Nilotinib inhibits skeletal muscle cell differentiation, particularly the myoblast-to-myotube transition, by decreasing the levels of myogenin and MyoD and reducing myotube formation. Moreover, we found that Nilotinib induces myoblast proliferation, causing impairments on myoblasts cell-cycle withdrawal through the activation of ERK1/2 and AKT pathways. Finally, we found that p38 MAPK is a new off-target of Nilotinib, inhibiting p38 phosphorylation. Since TKIs have also been shown to be an efficient treatment for attenuating muscle pathology in several MD mice models, it is necessary to investigate the long-term effects of TKIs on skeletal muscle homeostasis, along with the potential detrimental effects on cell differentiation and proliferation in cancer patients receiving TKI therapies.

## Additional files

Additional file 1: Figure S6. Morphology of mouse myogenic cultures. Phase micrographs showing the morphology of primary adult mouse myogenic cultures seeded on gelatin-coated dishes. The cells were isolated by Collagenase/Dispase digestion, and cultures were maintained in rich growth medium according to the protocol detailed in this paper. Proliferating cells (rounded ones) were observed in early cultures. Multinucleated myotubes can already be observed from days 3–4. Images were taken with a 10× objective. (PDF 3922 kb)

Additional file 2: Figure S1. Pax7 levels are unaffected by Nilotinib treatment during skeletal muscle differentiation. (A) Representative western blot that evaluates Pax7 levels during a 6-day time-course of skeletal muscle differentiation. GAPDH was used as the loading control. The lower panel shows the quantification of six independent experiments to evaluate Pax7 expression. The values correspond to the mean ± SEM. *n.s* non-significant, *n = 6*; one-way ANOVA with Bonferroni post-test. (PDF 160 kb)

Additional file 3: Figure S2. Nilotinib does not cause apoptosis of C2C12 myoblasts. (A) General gating using FSC (linear scale) and SSC (linear scale) to identify cell populations. C2C12 myoblasts were grown in GM and treated with Nilotinib 5 μM for 24 h (right panel) or DMSO (left panel). Cells were then treated with 7-AAD and analyzed by flow cytometry. (B) Note that the Nilotinib treatment does not change the number of apoptotic cells, determined by 7-AAD-fluorescence intensity. (PDF 116 kb)

Additional file 4: Figure S3. p38, ERK1/2, and AKT phosphorylation during C2C12 skeletal myogenesis. (A) Representative Western blots evaluating the phosphorylation of p38, ERK1/2, and AKT during a 6-day time curse of skeletal myogenesis using the C2C12 myoblast cell line. The total content of these proteins was used as loading controls. MyHC expression was used as a positive control of skeletal myogenesis. (PDF 486 kb)

Additional file 5: Figure S4. Nilotinib inhibits p38 but activates ERK1/2 and AKT in myoblasts. (A) Representative western blot of myoblasts, evaluating ERK1/2 and AKT phosphorylation using U0126 and LY294002 inhibitors along with Nilotinib. Nilotinib treatment (5 μM) was performed for 1 h. Total ERK1/2 and AKT were used as loading controls. (B) Western blot analyses of two representative experiments, evaluating the phosphorylation of FAK (Tyr397) and SAPK/JNK (Thr183/Tyr185) in myoblasts after Nilotinib treatment for 1 h. Total FAK and GAPDH were used as loading controls. (PDF 464 kb)

Additional file 6: Supplementary methods. (DOCX 1691 kb)

Additional file 7: Figure S5. Nilotinib induces myoblast proliferation and histone 3 phosphorylation. (A) Quantification of survival/proliferation using the MTT assay. C2C12 myoblast cultures were treated with different concentrations of Nilotinib in DM for 24 h. **P < 0.005, ***P < 0.0001; *n* = 3; one-way ANOVA with Bonferroni post-test. (B) Western blot analysis to evaluate phosphorylation of histone 3 (Ser28) after Nilotinib treatment in combination with UO126 and LY294002 inhibitors in differentiation medium for 24 h. Tubulin was used as the loading control. (PDF 161 kb)

## Abbreviations

(Ara-C): Cytosine β-D-arabinofuranoside
BrdU: 5-Bromo-2′-deoxyuridine
CML: Chronic myelogenous leukemia
DDRs: Discoidin domain receptors
FAK: Focal adhesion kinase
FAPs: Fibro/adipogenic progenitors
MAPK: Mitogen-activated protein kinase
MD: Muscular dystrophies
MRFs: Muscle regulatory factors
MuSCs: Muscle stem cells
PDGFRα: Platelet-derived growth factor receptor alpha
TKIs: Tyrosine kinase inhibitors
