# Supplementary figures and images for: Nilotinib impairs skeletal myogenesis by increasing myoblast proliferation
Source: Skelet Muscle. 2018 Feb 20;8:5. doi: 10.1186/s13395-018-0150-5 (PMC5819301; doi:10.1186/s13395-018-0150-5)

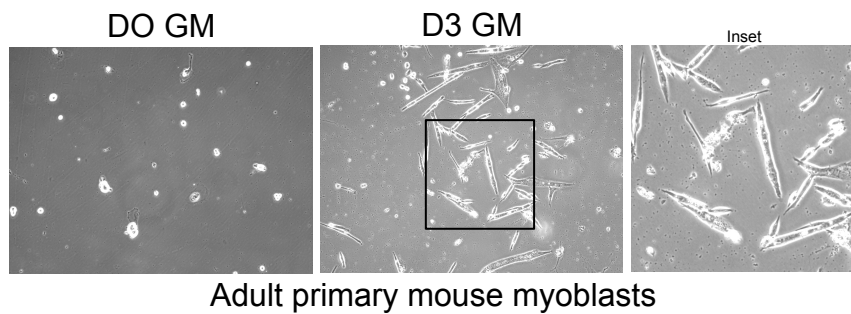

Fig S6.

Supplement: Supplementary file 1 — Morphology of mouse myogenic cultures. Phase micrographs showing the morphology of primary adult mouse myogenic cultures seeded on gelatin-coated dishes. The cells were isolated by Collagenase/Dispase digestion, and cultures were maintained in rich growth medium according to the protocol detailed in this paper. Proliferating cells (rounded ones) were observed in early cultures. Multinucleated myotubes can already be observed from days 3–4. Images were taken with a 10× objective. (PDF 3922 kb) [file 13395_2018_150_MOESM1_ESM.pdf]

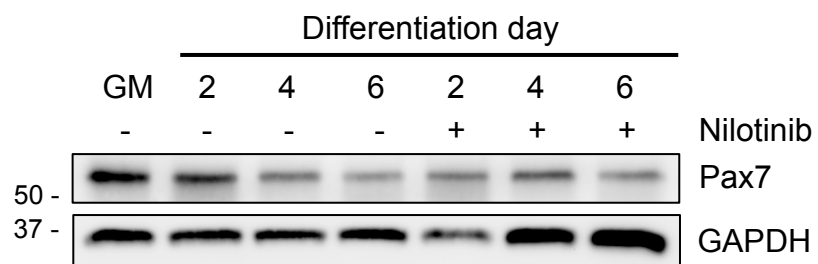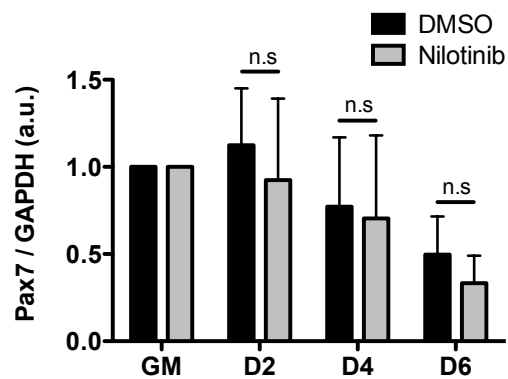

Fig S1.

Supplement: Supplementary file 2 — Pax7 levels are unaffected by Nilotinib treatment during skeletal muscle differentiation. (A) Representative western blot that evaluates Pax7 levels during a 6-day time-course of skeletal muscle differentiation. GAPDH was used as the loading control. The lower panel shows the quantification of six independent experiments to evaluate Pax7 expression. The values correspond to the mean ± SEM. n.s non-significant, n = 6; one-way ANOVA with Bonferroni post-test. (PDF 160 kb) [file 13395_2018_150_MOESM2_ESM.pdf]

A

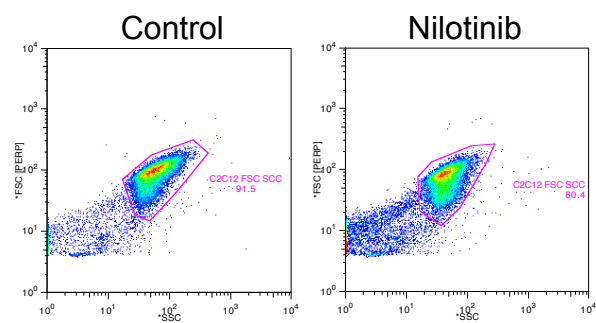

B

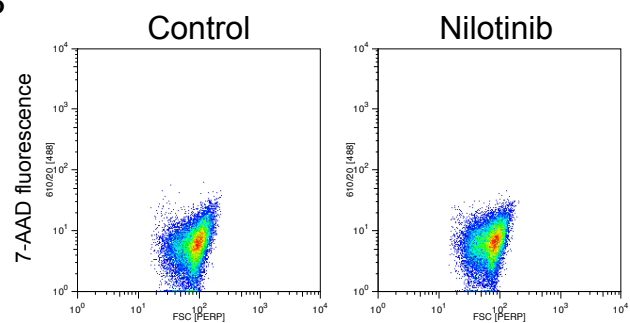

Fig S2.

Supplement: Supplementary file 3 — Nilotinib does not cause apoptosis of C2C12 myoblasts. (A) General gating using FSC (linear scale) and SSC (linear scale) to identify cell populations. C2C12 myoblasts were grown in GM and treated with Nilotinib 5 μM for 24 h (right panel) or DMSO (left panel). Cells were then treated with 7-AAD and analyzed by flow cytometry. (B) Note that the Nilotinib treatment does not change the number of apoptotic cells, determined by 7-AAD-fluorescence intensity. (PDF 116 kb) [file 13395_2018_150_MOESM3_ESM.pdf]

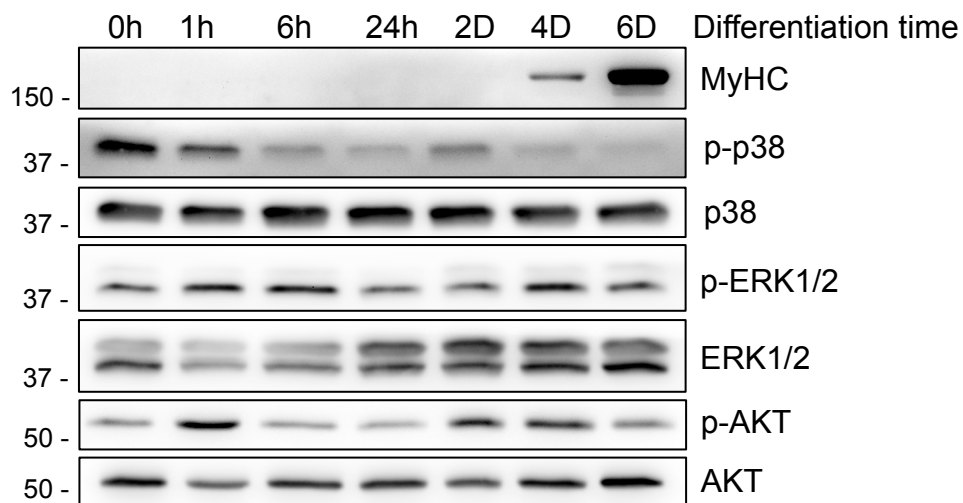

Fig S3.

Supplement: Supplementary file 4 — p38, ERK1/2, and AKT phosphorylation during C2C12 skeletal myogenesis. (A) Representative Western blots evaluating the phosphorylation of p38, ERK1/2, and AKT during a 6-day time curse of skeletal myogenesis using the C2C12 myoblast cell line. The total content of these proteins was used as loading controls. MyHC expression was used as a positive control of skeletal myogenesis. (PDF 486 kb) [file 13395_2018_150_MOESM4_ESM.pdf]

A

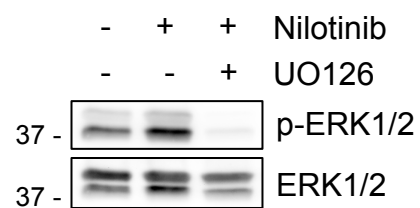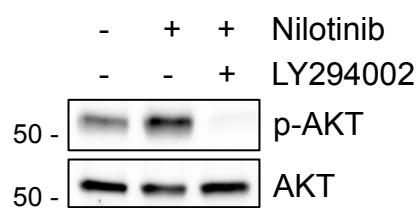

B

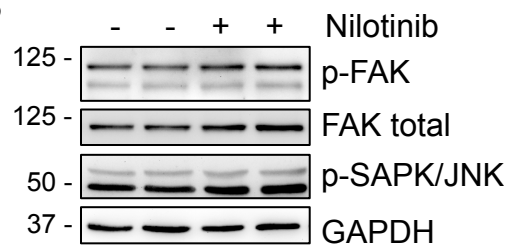

Fig S4.

Supplement: Supplementary file 5 — Nilotinib inhibits p38 but activates ERK1/2 and AKT in myoblasts. (A) Representative western blot of myoblasts, evaluating ERK1/2 and AKT phosphorylation using U0126 and LY294002 inhibitors along with Nilotinib. Nilotinib treatment (5 μM) was performed for 1 h. Total ERK1/2 and AKT were used as loading controls. (B) Western blot analyses of two representative experiments, evaluating the phosphorylation of FAK (Tyr397) and SAPK/JNK (Thr183/Tyr185) in myoblasts after Nilotinib treatment for 1 h. Total FAK and GAPDH were used as loading controls. (PDF 464 kb) [file 13395_2018_150_MOESM5_ESM.pdf]

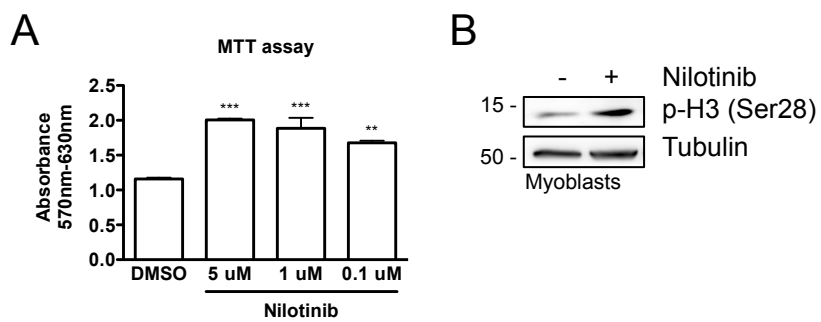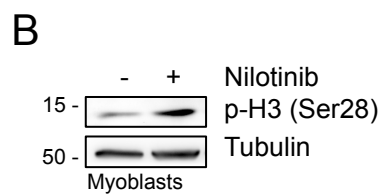

Fig S5.

Supplement: Supplementary file 7 — Nilotinib induces myoblast proliferation and histone 3 phosphorylation. (A) Quantification of survival/proliferation using the MTT assay. C2C12 myoblast cultures were treated with different concentrations of Nilotinib in DM for 24 h. **P < 0.005, ***P < 0.0001; n = 3; one-way ANOVA with Bonferroni post-test. (B) Western blot analysis to evaluate phosphorylation of histone 3 (Ser28) after Nilotinib treatment in combination with UO126 and LY294002 inhibitors in differentiation medium for 24 h. Tubulin was used as the loading control. (PDF 161 kb) [file 13395_2018_150_MOESM7_ESM.pdf]
