# Supplementary material for: Nilotinib impairs skeletal myogenesis by increasing myoblast proliferation
Source: Skelet Muscle. 2018 Feb 20;8:5. doi: 10.1186/s13395-018-0150-5 (PMC5819301; doi:10.1186/s13395-018-0150-5)
Supplement: Supplementary file 6 — Supplementary methods. (DOCX 1691 kb) [file 13395_2018_150_MOESM6_ESM.docx]

**Supplementary methods**

**7-AAD-flow cytometry analysis**

For 7-AAD viability analyses, cells were grown in growth medium and treated for 24 h with DMSO or Nilotinib. Then, myoblasts were washed 3 times with PBS 1X prior to the addition of cell dissociation buffer (0.025% Trypsin-EDTA (0.5 X) (Life technologies, NY, USA) plus (0.5% BSA, 1 mM EDTA, 1 mM EGTA, pH 7.4). Cells were incubated for 15 minutes until they detached from the plate. To evaluate apoptosis 7-AAD was added (1:500) to the detached-cells (1x10^6^ cells) and incubated for 15 minutes in the dark, prior to analysis by flow cytometry. The analysis was performed on a BD Influx flow cytometer (Becton Dickinson, USA) using 488-excitation laser. For each sample, 20,000 events were collected and analyzed. Gating and the percentage of cells existing within gates was determined using FlowJo 8.7 software (Ashland, OR, USA).

**Colorimetric MTT (tetrazolium) assay**

MTT (3-(4,5-dimethylthiazol-2-yl)-2,5-diphenyl tetrazolium bromide (#475989; Calbiochem, USA) was dissolved in PBS (5 mg/ml) and filtered. 20 h after Nilotinib treatment, stock MTT solution (10X) was added to all assay wells, and plates were incubated at 37°C for 4 h. Warm lysis buffer (50% N,N-Dimethylformamide + 20% SDS in destilled water) was added to all wells and mixed thoroughly to dissolve the dark blue crystals. After a few minutes at room temperature to ensure that all crystals were dissolved, the plates were read in a Synergy H1 equipment using the software Gen5 (BioTek, USA), using a test wavelength of 570 nm, and a reference wavelength of 630 nm. Plates were normally read within 1 h of adding the lysis solution.
